# The Impact of COVID-19 Quarantine on Patients With Dementia and Family Caregivers: A Nation-Wide Survey

**DOI:** 10.3389/fnagi.2020.625781

**Published:** 2021-01-18

**Authors:** Innocenzo Rainero, Amalia C. Bruni, Camillo Marra, Annachiara Cagnin, Laura Bonanni, Chiara Cupidi, Valentina Laganà, Elisa Rubino, Alessandro Vacca, Raffaele Di Lorenzo, Paolo Provero, Valeria Isella, Nicola Vanacore, Federica Agosta, Ildebrando Appollonio, Paolo Caffarra, Cinzia Bussè, Renato Sambati, Davide Quaranta, Valeria Guglielmi, Giancarlo Logroscino, Massimo Filippi, Gioacchino Tedeschi, Carlo Ferrarese, the SINdem COVID-19 Study Group, Erica Gallo

**Affiliations:** Aging Brain and Memory Clinic, Department of Neuroscience, University of Torino, Turin, Italy; Regional Neurogenetic Centre ASP-CZ Catanzaro, Catanzaro, Italy; Memory Clinic, Fondazione Policlinico Agostino Gemelli, IRCCS Universitá Cattolica del Sacro Cuore, Rome, Italy; Department of Neuroscience, University of Padua, Padua, Italy; CDCD Aulss6 Alta Padovana, Padua, Italy; Department of Biotechnological and Applied Clinical Sciences, Neurological Institute, University of L’Aquila, L’Aquila, Italy; Avezzano Hospital, Avezzano, Italy; Department of Neuroscience, Imaging and Clinical Sciences, University G. d’Annunzio, Chieti, Italy; UOC Neurology, G. Mazzini Hospital, Teramo, Italy; Clinica Neurologica San Salvatore Hospital, L’Aquila, Italy; Division of Neurology, Scientific Institute for Research, Hospitalization, and Care (IRCCS), Foundation “Carlo Besta” Neurological Institute, Milan, Italy; CDCD Serra Spiga ASP Cosenza, Cosenza, Italy; CDCD Polistena Laureana ASP Reggio Calabria, Cinquefrondi, Italy; CDCD Jonio Sud District ASP Cosenza, Corigliano-Rossano, Italy; First Division of Neurology, University of Campania “Luigi Vanvitelli”, Napoli, Napoli, Italy; CDCD AORN “Ospedale dei Colli” – CTO, Napoli, Napoli, Italy; ASL Napoli 3 Sud, Napoli, Italy; CDCD Neurologia, University of Campania “Federico II”, Napoli, Italy; IRCCS Istituto delle Scienze Neurologiche di Bologna, UOC Clinica Neurologica Rete Neurologica Metropolitana (NEUROMET), Italy; Department of Neuroscience, Neurology Unit, AOU Sant’Anna di Icona – Ferrara, Ferrara, Italy; CDCD Ospedale del Delta, AUSL Ferrara, Ferrara, Italy; CDCD, AUSL of Parma, Parma, Italy; AUO Policlinico Modena, Modena, Italy; UOC Cognitive Disorders and Dementia, Department of Primary Care, AUSL Modena, Italy; Fondazione Santa Lucia IRCCS, Roma, Italy and Menninger Department of Psychiatry and Behavioural Sciences, Baylor College of Medicine, Houston, TX, United States; Fondazione Santa Lucia IRCCS, Roma, and Dipartimento di Neuroscienze, Università di Roma “Tor Vergata”, Roma, Italy; Campus Biomedico, University of Roma, Roma, Italy; Department of Neuroscience, University of Roma “La Sapienza”, Roma, Italy; Department of Geriatrics, Fondazione Poliambulanza di Brescia, Italy; Luigi Sacco Hospital, University of Milano, Milano, Italy; Unit of Neurology, IRCCS San Raffaele Scientific Institute and Vita-Salute San Raffaele University, Milan, Italy; Unit of Behavioral Neurology IRCCS Mondino Foundation, and Department of Brain and Behavioral Sciences, University of Pavia, Italy; Department of Experimental and Clinical Medicine, Polytechnic University of Marche, Ancona, Italy; CDCD Mazzoni Hospital, Ascoli Piceno, Italy; Fondazione Santa Lucia IRCCS, Roma, Italy; AOU Sant’Andrea, Roma, Italy; AO San Giovanni Addolorata, Roma, Italy; CDCD Area Vasta 4, Fermo, Italy; Geriatric Operative Unit, IRCCS-INRCA, Fermo, Italy; CDCD Area Vasta 3, Macerata, Italy; Azienda Ospedaliera Marche Nord, Pesaro, Italy; CDCD San Benedetto del Tronto, Italy; CDCD IRCSS Neuromed di Pozzilli, Isernia, Italy; Department of Neuroscience, Neurology Division, OORR Foggia, Italy; Neurodegenerative Centre, University of Bari “Aldo Moro”, Bari, Italy; CDCD DSS of Campi Salentina, Lecce, Italy; CDCD DSS of Lecce, Lecce, Italy; CDCD DSS of Maglie, Maglie, Italy; Department of Neurology, University of Milano – Bicocca, Milano, Italy; CDCD PO Santissima Trinità, ASSL Cagliari, Cagliari, Italy; CDCD Area Vasta 1, Cagliari, Italy; Section of Neurology, Department of Biomedicine, Neuroscience and Advanced Diagnostics, University of Palermo, Palermo, Italy; Neurology and Neurophysiopathology Unit, AOUP “Paolo Giaccone”, Palermo, Italy; UO Neurodegenerative Disorders, ASP 2, Caltanisetta, Italy; Department “G.F. Ingrassia”, University of Catania, Catania, Italy; AO Cannizzaro, Catania, Italy; Psychogeriatric Unit, ASP Messina, Messina, Italy; Neurology I, Department of Neuroscience, Psychology, Drug Research and Child Health, AOU Careggi, Firenze, Italy; CDCD, Neurology I, AOU University of Pisa, Pisa, Italy; Geriatric Unit, Department of Clinical and Experimental Medicine, University of Pisa, Pisa, Italy; AOU Careggi and University of Florence; CDCD Territoriale, USL Umbria 1, Perugia, Italy; Department of Medicine, University of Perugia, Perugia, Italy; CDCD AUSSL 7 Pedemontana, Bassano del Grappa, Italy; CDCD Geriatria, Dolo, Venezia, Italy; CDCD Geriatric Unit, University of Padua, Padua, Italy; CDCD AULSS 9 Scaligera, Verona, Italy; CDCD AULSS 2 Marca Trevigiana, Treviso, Italy; ASST Grande Ospedale Metropolitano, Niguarda, Milano, Italy; ^1^Aging Brain and Memory Clinic, Department of Neuroscience “Rita Levi Montalcini”, University of Torino, Turin, Italy; ^2^Department of Neuroscience and Mental Health, AOU Città della Salute e della Scienza di Torino, Turin, Italy; ^3^Regional Neurogenetic Centre, Department of Primary Care, ASP-CZ, Catanzaro, Italy; ^4^Memory Clinic, Fondazione Policlinico Agostino Gemelli, IRCCS, Università Cattolica del Sacro Cuore, Rome, Italy; ^5^Department of Neuroscience, University of Padua, Padua, Italy; ^6^Department of Neuroscience, Imaging and Clinical Sciences, University G. d’Annunzio of Chieti–Pescara, Chieti, Italy; ^7^CDCD Ospedale del Delta, AUSL Ferrara, Ferrara, Italy; ^8^Department of Neuroscience “Rita Levi Montalcini”, University of Torino, Turin, Italy; ^9^Center for Omics Sciences, IRCCS S. Raffaele Scientific Institute, Milan, Italy; ^10^Department of Medicine and Surgery and Milan Center for Neuroscience (NeuroMi), University of Milano–Bicocca, Monza, Italy; ^11^National Institute of Health, Rome, Italy; ^12^Neuroimaging Research Unit, Division of Neuroscience, IRCCS San Raffaele Scientific Institute, Milan, Italy; ^13^Neurology Unit, Neurorehabilitation Unit, and Neurophysiology Service, IRCCS San Raffaele Scientific Institute, Vita-Salute San Raffaele University, Milan, Italy; ^14^Unit of Neuroscience, University of Parma, Parma, Italy; ^15^Department of Clinical Research in Neurology, Center for Neurodegenerative Diseases and the Aging Brain, University of Bari, Bari, Italy; ^16^Department of Basic Medicine, Neuroscience, and Sense Organs, University of Bari Aldo Moro, Bari, Italy; ^17^Department of Medical and Surgical Sciences, University of Campania “L. Vanvitelli”, Naples, Italy

**Keywords:** quarantine, COVID-19, dementia, Alzheimer’s disease, BPSD, caregiver burden

## Abstract

**Introduction:**

Previous studies showed that quarantine for pandemic diseases is associated with several psychological and medical effects. The consequences of quarantine for COVID-19 pandemic in patients with dementia are unknown. We investigated the clinical changes in patients with Alzheimer’s disease and other dementias, and evaluated caregivers’ distress during COVID-19 quarantine.

**Methods:**

The study involved 87 Italian Dementia Centers. Patients with Alzheimer’s Disease (AD), Dementia with Lewy Bodies (DLB), Frontotemporal Dementia (FTD), and Vascular Dementia (VD) were eligible for the study. Family caregivers of patients with dementia were interviewed by phone in April 2020, 45 days after quarantine declaration. Main outcomes were patients’ changes in cognitive, behavioral, and motor symptoms. Secondary outcomes were effects on caregivers’ psychological features.

**Results:**

4913 patients (2934 females, 1979 males) fulfilled the inclusion criteria. Caregivers reported a worsening in cognitive functions in 55.1% of patients, mainly in subjects with DLB and AD. Aggravation of behavioral symptoms was observed in 51.9% of patients. In logistic regression analysis, previous physical independence was associated with both cognitive and behavioral worsening (odds ratio 1.85 [95% CI 1.42–2.39], 1.84 [95% CI 1.43–2.38], respectively). On the contrary, pandemic awareness was a protective factor for the worsening of cognitive and behavioral symptoms (odds ratio 0.74 [95% CI 0.65–0.85]; and 0.72 [95% CI 0.63–0.82], respectively). Approximately 25.9% of patients showed the onset of new behavioral symptoms. A worsening in motor function was reported by 36.7% of patients. Finally, caregivers reported a high increase in anxiety, depression, and distress.

**Conclusion:**

Our study shows that quarantine for COVID-19 is associated with an acute worsening of clinical symptoms in patients with dementia as well as increase of caregivers’ burden. Our findings emphasize the importance to implement new strategies to mitigate the effects of quarantine in patients with dementia.

## Introduction

The coronavirus disease 2019 (COVID-19) pandemic is, nowadays, a global public health emergency (Palacios Cruz et al., 2020). By December 8, 2020, over 66 million confirmed cases of COVID-19 with more than 1.530.000 deaths worldwide have been reported to the World Health Organization. Older adults and patients with certain comorbidities, many of whom are of advanced age, are particularly susceptible to more severe consequences of the disease (Shahid et al., 2020). The impact of the pandemic to the healthcare systems has been disruptive, and prevention as well as treatment services for patients with non-communicable diseases have been severely reduced.

Trying to slow down the spread of pandemic, several governments launched mitigation strategies, based mainly on quarantine. Quarantine is efficacious in reducing incidence and mortality during outbreaks of infectious diseases, and preliminary indications suggest that this strategy is effective also in the COVID-19 pandemic (Nussbaumer-Streit et al., 2020). However, home confinement is an unpleasant experience, due to the significant limitations in physical, cognitive, and social activities. Studies related to the 2003 outbreak of severe acute respiratory syndrome (SARS) in China and Canada, as well as the 2014 Ebola outbreak in Africa, showed that quarantine is associated with several negative psychological effects, like depression, irritability, anger, and insomnia (Hawryluck et al., 2004; Barbisch et al., 2015; Brooks et al., 2020; Gualano et al., 2020). Deaths by suicide increased in older adults during the SARS epidemic in Hong Kong (Cheung et al., 2008). In addition, long-lasting psychiatric effects of quarantine have been reported (Mak et al., 2009).

Individuals with Alzheimer’s disease and other dementias are among the most vulnerable persons in the society, and the COVID-19 outbreak is likely to have further exacerbated their frailty (Wang et al., 2020). Quarantine effects in dementia patients and family caregivers have never been adequately investigated. All the negative effects of quarantine previously reported may be exacerbated in individuals with dementia, whose physical and social isolation may amplify the functional limitations and the pre-existing conflicts within the family. Patients with dementia living at home significantly depend on family caregivers for assistance, and caring for dementia patients causes a significant burden in caregivers (Chiao et al., 2015; Raggi et al., 2015; Brown et al., 2020). Higher levels of cognitive, behavioral and motor impairment in patients with dementia are associated with greater burden and distress in their caregivers (Black et al., 2018; Yang et al., 2019; Xiong et al., 2020). Therefore, there is an urgent need to investigate clinical and psychological changes due to quarantine in patients with dementia and their caregivers.

This study aimed at evaluating the effects of quarantine in Italian patients with different types and severity of dementia and their caregivers. Approximately 45 days after quarantine declaration by the Italian government, we interviewed the family caregivers of persons with dementia referring to several Italian Centers for Cognitive Disorders and Dementia (CDCDs), investigating patients’ variations in cognitive, behavioral, and motor symptoms. Besides, we evaluated the changes in prescribed medications, caregiver’s burden, and changes in health services provided by the Italian National Health Service.

## Materials and Methods

### Study Design, Centers and Participants

This is a multicentric, nation-wide survey. Eighty-nine Italian CDCDs were initially recruited for the study. First of all, a semi-structured questionnaire (see Supplementary Material) was administered to the Director of each Centre in order to evaluate its qualitative and quantitative characteristics, and variations in clinical activities after quarantine declaration. Due to the quarantine rules, all the out-patients visits were stopped. Therefore, the clinical staff members of each CDCD consecutively contacted by phone the family caregivers of patients registered in the waiting list. After an oral consent, a semi-structured, self-made interview gathering demographic and clinical data on the patient and the caregiver was administered (see Supplementary Material). Inclusion criteria were a diagnosis of one of the most common forms of dementias including: (1) Alzheimer’s Disease (AD), (2) Dementia with Lewy Bodies (DLB), (3) Frontotemporal Dementias (FTD), and (4) Vascular Dementia (VD). Exclusion criteria included current or previous diagnosis of other forms of dementias, mild cognitive impairment, and subjective cognitive complaints.

### Description of the Survey

We collected all the socio-demographic characteristics of patients. The stage of dementia was assessed using the Clinical Dementia Rating (CDR) (Morris et al., 1997) scale. Furthermore, we investigated the patient’s variation in his/her clinical status, analyzing cognitive, psychological, behavioral, and motor symptoms during the quarantine period. Briefly, the following cognitive features were investigated: changes in memory, spatial and temporal orientation, language, attention, and perception. Questions about patient’s awareness of the pandemic were administered. In addition, we acquired data regarding variations in behavioral and psychological symptoms of dementia (BPSD, i.e., irritability, apathy, agitation, anxiety, depression, sleep change, aggressiveness, wandering, appetite change, hallucinations, and delusions), reporting for these symptoms both a worsening and/or a new onset that occurred during the quarantine period. Further questions about the need for modifying therapy because of BPSD changes were administered. Finally, we investigated the changes in patients’ motor activity, evaluating through a 5-point ordinal scale specific question if the subject walked better from the beginning of the quarantine period, remained stable, walked slower, became wheelchair, or became confined to bed.

### Caregivers’ Socio-Demographic Characteristics

Caregivers’ were asked about the cohabitation with the patient, composition of the family, and own work activity. Other questions concerned the impact of the quarantine period on caring for a person with dementia on their caregivers’ lives, investigating both social and psychological effects (changes in caregiver’s life, change in the relationship with the patient, concern for the pandemic, changes in therapeutic assistance, need to seek help from the emergency department, use of telemedicine, need to support during quarantine, own feeling of depression, anxiety, irritability, distress, overwhelm, and abandonment). Further details are available at Supplementary Material.

### Statistical Analysis

We performed statistical analysis using SPSS software, version 21, and the R statistical computing environment, version 3.6.2 (IBM Corp, 2012; R Core Team, 2013). Due to the low rate of missing data (<1%) no imputation was made. Firstly, we performed a descriptive analysis of all the demographic and clinical data. Then, we performed univariable and multivariable logistic regression of the dependent variables (outcomes in clinical symptoms, all described by binary variables) on the collected independent variables, using mixed effects logistic regression (as implemented in the lme4 R package) with the center as a random effect and all other regressors as fixed effects. Regressors with significant *p* (<0.05) in univariable logistic regression were included in multivariable regression. Bonferroni correction was applied to all the *p*-values of multivariable analysis, considering all outcomes together, as multivariable analysis is considered as our final result. We controlled for caregiver stress by including it in the multivariable analysis as a confounder, as a numerical fixed effect. The level of statistical significance was set at *p* < 0.05.

### Ethical Standards

The study was initially approved by the Ethics Committee of the Coordinating Centre (University of Torino on April 7, 2020, n.00150/2020) and then by the local ethics boards. Caregivers gave first oral and then written fully informed consent to the study.

## Results

### Changes in Health Services

**FIGURE 1:**
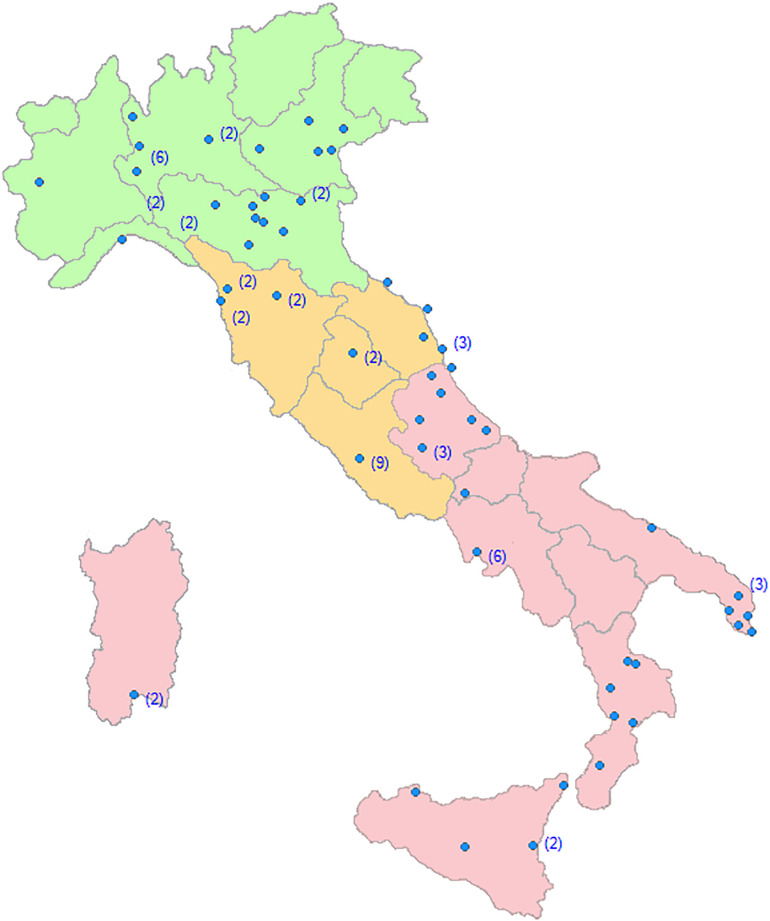
Regional distribution of the Dementia Centers involved in the SINdem COVID-19 study. Within brackets, the number of Centers in each city.

The response rate of CDCDs to the proposed questionnaires was 98%. Only two CDCDs were excluded because they did not recruit patients. The final 87 recruiting Centers were homogeneously distributed throughout the Italian territory (Figure 1).

Thirty-two percent of patients with dementia were recruited in Northern, 32% in Central, and 36% in Southern Italy. Thirty percent of CDCDs was based at university hospitals, 34% to general hospitals, and 36% to territorial based health services. In the pre-COVID-19 pandemic period, each Center followed a mean of 160 dementia patients per month. After the quarantine declaration, 85% of the CDCD suspended medical and psychological appointments, and visits were restricted to emergencies. Different forms of telemedicine (from phone calls to videoconferencing) were activated in 78% of Centers. One out of two Center provided on-line psychological support for caregivers. All the randomized clinical trials were stopped. Finally, 94% of support activities for dementia patients (Alzheimer’s café, Day Center, etc.) were closed.

### Caregivers

The Survey response rates of the family caregivers ranged from 91.3 to 99.1%, according to different dementia Centers. We interviewed 5321 caregivers of patients regularly followed at different CDCDs. After data cleaning, the information collected from 4913 family caregivers (women 2934, mean age ± *SD* = 58.2 ± 12.0 years; men 1979, mean age ± *SD* = 60.7 ± 13.9 years) were analyzed. Fifty-nine % were cohabitants with the patients, and 36% were spouses of the dementia care recipient. Approximately half of the caregivers reported that quarantine induced a significant change in their lifestyle, with 30.3% complaining a reduction in time devoted to their own activities, and 15.5% reporting an increase in intrafamilial psychological conflicts. Caregivers reported a significant increase in anxiety (45.9%), depression (18.6%), irritability (26.2%), and distress (28.9%). Finally, 80.8% of the caregivers reported that telemedicine has been of help during quarantine.

### Dementia Patients

From April 14 to April 27, 2020, we collected data regarding 4913 patients with dementia (2934 females, mean age ± *SD* = 78.9 ± 8.2 years; 1979 males, mean age ± *SD* = 77.2 ± 8.0 years) regularly followed at CDCDs. Demographic and clinical characteristics of dementia patients are shown in Table 1.

**TABLE 1:** Demographic and clinical characteristics of study participants.

**Patients**	**Total (*n* = 4913)**	**AD (*n* = 3372)**	**DLB (*n* = 360)**	**FTD (*n* = 415)**	**VD (*n* = 766)**
Age (years, mean ± SD)	78.3 ± 8.2	78.3 ± 8.0	78 ± 7.3	72.3 ± 8.9	81.6 ± 7.0
Sex (female, %)	59.7	63.5	42.2	46.7	58.4
Duration of the disease (years, mean ± SD)	4.5 ± 3.1	4.6 ± 3.1	4.5 ± 3	4.8 ± 3.2	4.1 ± 2.9
**Regional distribution**					
North (%)	32.2	26.5	35.3	47.5	47.8
Centre (%)	31.5	34.1	36.4	21.2	23.4
South - Islands (%)	36.3	39.4	28.3	31.3	28.8
**CDR Stage (%)**					
1	25.0	24.3	26.3	23.4	28.4
2	47.8	49.2	41.9	48.6	43.8
3	27.2	26.5	31.8	28.0	27.8
Duration of the quarantine (days, mean ± SD)	47.2 ± 6.4	47.2 ± 6.5	46.6 ± 5.6	46.4 ± 5.3	47.8 ± 6.8
**Changes in cognitive symptoms (Yes, %)**	55.1	55.7	59.6	48.3	54.2
Sex (female, %)	58.4	63.0	36.3	44.7	55.2
**Changes in BPSD (Yes, %)**	51.9	50.5	63.8	55.3	50.3
Sex (female, %)	57.9	62.9	38.4	45.4	55.1
**New onset BPSD (Yes, %)**	25.9	26.7	23.3	21.9	25.6
Sex (female, %)	56.7	59.8	41.7	41.8	56.1
**Changes in motor symptoms (Yes, %)**	36.7	33.2	52.8	40.2	42.3
Sex (female, %)	58.6	65.0	44.7	43.1	52.5
**Caregivers**					
Age (years, mean ± SD)	59.3 ± 13	59.3 ± 13.1	60.7 ± 12.7	59.1 ± 13.6	60 ± 12.4
Sex (female, %)	53.9	51.2	66.4	55.4	59.4
Cohabitant caregiver (%)	58.9	58.1	63.5	69.6	54.4
**Degree of kinship**					
Spouses (%)	36.0	35.0	43.1	54.8	26.9
Son/daughter (%)	54.5	55.5	48.7	37	62.5
Others (%)	9.5	9.5	8.2	8.2	10.6
Increase in anxiety (%)	45.9	46.1	43.4	44.2	47.4
Increase in depression (%)	18.6	17.2	21.3	24.3	20.3

*AD, Alzheimer’s disease; DLB, Dementia with Lewy Bodies; FTD, frontotemporal dementia; VD, vascular dementia; CDR, Clinical Dementia Rating; BPSD, Behavioral and Psychological symptoms of dementia.*

Family caregivers reported that, after a quarantine period of approximately 47 days, dementia patients showed a worsening in cognitive symptoms (+55.1%), and behavioral symptoms (+51.9%), the onset of new behavioral symptoms (+25.9%), and an increase in motor symptoms (+36.7%). According to caregivers, 40.1% of patients were totally aware of quarantine declaration, 36.0% were only partially aware, while 23.9% were totally unaware. Awareness of the quarantine varied significantly among the dementia subgroups, with DLB patients showing the highest degree (69.8%) of partial of total unawareness of pandemic.

### Changes in Cognitive Symptoms

**FIGURE 2:**
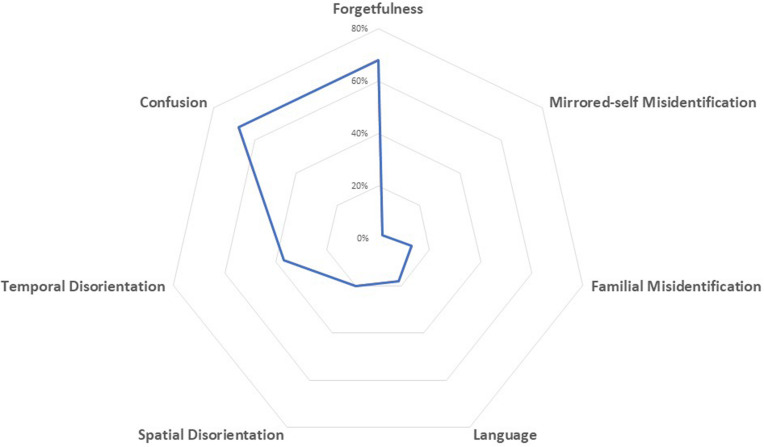
Spider-plot of changes in cognitive function during quarantine; dots mark the mean proportion of each symptom.

Caregivers reported a worsening in cognitive symptoms in approximately 60% of dementia participants. About 37% of dementia participants showed worsening in two or more cognitive domains. Deficits more often reported as increased were: forgetfulness (68%), confusion (67.9%), and temporal disorientation (37%). Figure 2 displays the frequency distribution of cognitive symptoms worsened during quarantine.

When we examined diagnostic subgroups, we found a worsening of cognitive symptoms mainly in patients with DLB (59.6%) and AD (55.7%), followed by VD (54.2%), and FTD (48.3%) patients. Thirty-eight % of patients with AD showed a worsening in memory functions, 37.5% presented an increase in confusion, and 21.1% showed a worsening in temporal disorientation. The same effects were observed in patients with DLB, showing a worsening in memory functions in 26.1%, an increase in confusion in 40.3%, and in temporal disorientation in 20.3%. Patients with FTD showed mainly a worsening in language functions (25.5%). After multivariable analysis, the increase of cognitive symptoms in the overall group of patients with dementia was not associated with age, gender, duration of quarantine, disease severity, and changes in caregivers’ psychological features. Contrariwise, an inverse relationship between the aggravation of cognitive symptoms and the duration of the dementia was found (*p* < 0.001).

Interestingly, the pre-existing total or partial independence in motor function represents a risk factor for the worsening of cognitive functions (OR 1.85 [95% CI 1.42–2.39], *p* < 0.001; and OR 2.08 [95% CI 1.68–2.57], *p* < 0.001, respectively). Contrariwise, total awareness of quarantine was a protective factor against cognitive worsening (OR 0.68 [95% CI 0.57–0.81], *p* < 0.001) (Table 2).

**TABLE 2:** Univariable and multivariable analysis of risk and protective factors for cognitive and motor changes during quarantine in patients with dementia.

	**Univariable analysis**		**Multivariable analysis**		
	** Crude OR (95% CI)**	***P*-value**	**Adjusted OR (95% CI)**	***P*-value**	***P*-value after Bonferroni correction**
**Changes in cognitive symptoms**					
Age	1.00 (0.99 – 1.00)	0.24			
Sex (men vs. women)	1.15 (1.01 – 1.30)	0.03	1.13 (0.99 – 1.29)	0.07	1
Duration of the disease (years)	0.95 (0.93 – 0.97)	<0.001	0.94 (0.92 – 0.97)	<0.001	<0.001
Duration of quarantine (days)	1.00 (0.99 – 1.01)	0.97			
Disease severity – CDR 2	1.29 (1.11 – 1.50)	<0.001	1.19 (1.00 – 1.42)	0.05	1
Disease severity – CDR 3	1.02 (0.86 – 1.21)	0.81	0.93 (0.74 – 1.17)	0.52	1
Total physical independence	1.74 (1.40 – 2.16)	<0.001	1.85 (1.42 – 2.39)	<0.001	<0.001
Partial physical independence	2.19 (1.80 – 2.67)	<0.001	2.08 (1.68 – 2.57)	<0.001	<0.001
Total awareness of pandemic	0.74 (0.65 – 0.85)	<0.001	0.68 (0.57 – 0.81)	<0.001	<0.001
Partial awareness of pandemic	1.39 (1.18 – 1.64)	<0.001	1.20 (1.00 – 1.43)	0.05	1
**Changes in motor symptoms**					
Age	1.03 (1.02 – 1.04)	<0.001	1.02 (1.01 – 1.03)	<0.001	0.001
Sex (men vs. women)	1.11 (0.98 – 1.25)	0.12			
Duration of the disease (years)	1.05 (1.03 – 1.07)	<0.001	1.00 (0.98 – 1.03)	0.86	1
Duration of quarantine (days)	1.00 (0.99 – 1.01)	0.99			
Disease severity – CDR 2	1.94 (1.65 – 2.29)	<0.001	1.29 (1.07 – 1.56)	0.009	0.29
Disease severity – CDR 3	3.08 (2.56 – 3.70)	<0.001	1.60 (1.26 – 2.02)	<0.001	0.004
Total physical independence	0.32 (0.25 – 0.40)	<0.001	0.49 (0.37 – 0.64)	<0.001	<0.001
Partial physical independence	0.93 (0.77 – 1.12)	0.44	1.06 (0.86 – 1.30)	0.59	1
Total awareness of pandemic	0.44 (0.38 – 0.51)	<0.001	0.76 (0.64 – 0.91)	0.003	0.10
Partial awareness of pandemic	0.75 (0.64 – 0.88)	<0.001	0.93 (0.78 – 1.10)	0.39	1

*OR, Odds ratio; 95% CI, 95% confidence interval; CDR, Clinical Dementia Rating.*

### Changes in Behavioral Symptoms

**FIGURE 3:**
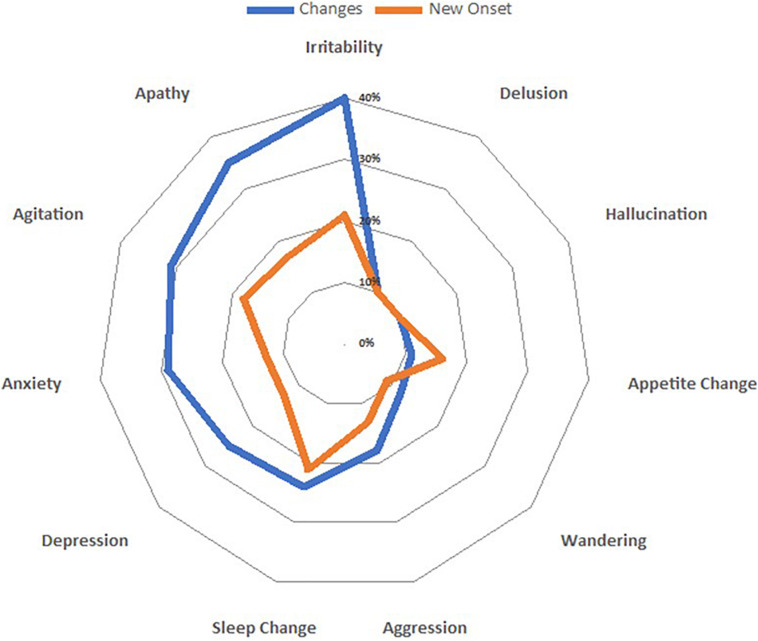
Spider-plot of variations in both clusters (changes and new onset) of behavioral and psychological symptoms; dots mark the mean increase of each behavior.

During the quarantine period, BPSD worsened in approximately half of the patients with dementia. The most frequently worsened BPSD were irritability (40%), followed by apathy (35%), and agitation (31%) (Figure 3).

Examining subgroups, we found an increase in the number of symptoms mainly in patients with DLB (63.8%), followed by FTD (55.3%), AD (50.5%), and VD (50.3%). The increase of neuropsychiatric symptoms in patients with dementia was associated with moderate disease severity (CDR 2, *p* = 0.009), but not remained significant after applying Bonferroni correction. The worsening of BPSD was associated with pre-existing total or partial autonomy in motor function (totally independent OR 1.84 [95% CI 1.43–2.38], *p* < 0.001; partially dependent OR 1.88 [95% CI 1.53–2.31], *p* < 0.001). After multivariate analysis, the worsening of BPSD was not associated with age, gender, duration of quarantine, disease severity, duration of disease, and changes in caregivers’ psychological features. On the contrary, total awareness of the pandemic was inversely related to worsening of BPSD (OR 0.75 [95% CI 0.63–0.89], *p* = 0.03) (Table 3).

**TABLE 3:** Univariable and multivariable analysis of risk and protective factors for increase in BPSD and onset of new BPSD during quarantine in patients with dementia.

	**Univariable analysis**		**Multivariable analysis**		
	**Crude OR (95% CI)**	***P*-value**	**Adjusted OR (95% CI)**	***P*-value**	***P*-value after Bonferroni correction**
**Increase in BPSD**					
Age	0.99 (0.98 – 0.99)	<0.001	0.99 (0.98 – 0.99)	0.005	0.16
Sex (men vs. women)	1.17 (1.04 – 1.32)	0.008	1.12 (0.99 – 1.28)	0.08	1
Duration of the disease (years)	0.98 (0.96 – 0.99)	0.04	0.97 (0.95 – 0.99)	0.01	0.48
Duration of quarantine (days)	0.99 (0.99 – 1.01)	0.66			
Disease severity – CDR 2	1.33 (1.15 – 1.54)	<0.001	1.26 (1.06 – 1.49)	0.009	0.30
Disease severity – CDR 3	1.32 (1.12 – 1.56)	0.001	1.21 (0.97 – 1.52)	0.09	1
Total physical independence	1.56 (1.26 – 1.93)	<0.001	1.84 (1.43 – 2.38)	<0.001	<0.001
Partial physical independence	1.90 (1.57 – 2.30)	<0.001	1.88 (1.53 – 2.31)	<0.001	<0.001
Total awareness of pandemic	0.72 (0.63 – 0.82)	<0.001	0.75 (0.63 – 0.89)	<0.001	0.03
Partial awareness of pandemic	1.25 (1.07 – 1.45)	0.005	1.20 (1.01 – 1.42)	0.04	1
**Onset of new BPSD**					
Age	1.00 (0.99 – 1.00)	0.32			
Sex (men vs. women)	1.19 (1.04 – 1.36)	0.01	1.10 (0.95 – 1.27)	0.21	1
Duration of the disease (years)	0.99 (0.97 – 1.01)	0.41			
Duration of quarantine (days)	1.00 (0.99 – 1.01)	0.72			
Disease severity – CDR 2	1.14 (0.96 – 1.35)	0.13			
Disease severity – CDR 3	1.08 (0.89 – 1.32)	0.41			
Total physical independence	1.69 (1.30 – 2.18)	<0.001	1.92 (1.45 – 2.55)	<0.001	<0.001
Partial physical independence	1.59 (1.26 – 2.02)	<0.001	1.55 (1.22 – 1.99)	<0.001	0.01
Total awareness of pandemic	0.78 (0.67 – 0.92)	0.003	0.74 (0.62 – 0.88)	<0.001	0.03
Partial awareness of pandemic	1.17 (0.99 – 1.39)	0.07	1.10 (0.92 – 1.32)	0.29	1

*BPSD, Behavioral and Psychological Symptoms of Dementia; OR, Odds ratio; 95% CI, 95% confidence interval; CDR, Clinical Dementia Rating.*

New BPSD occurred in approximately a quarter of the dementia patients. There was a different increase in the frequency of BPSD across groups. The most frequently reported new symptom was irritability (21.3%), followed by sleep change (21%), and agitation (18%) (Figure 2), which required therapy adjustments. The highest increase was observed in AD (26.7%), intermediate in VD and DLB (25.6 and 23.3%, respectively), and the lowest increase in FTD (21.9%). No suicide attempts were reported. The onset of new BPSD was associated with the total or partial physical independence (*p* < 0.001 and *p* = 0.01, respectively). Total awareness of the pandemic was inversely related to onset of new BPSD (OR 0.74, [95% CI 0.62–0.88], *p* = 0.03).

### Changes in Motor Symptoms

Increased motor dysfunction, mainly characterized by walking difficulties, was reported by caregivers in 36.7% of the patients. There was an increase mainly in patients with DLB (52.8%), followed by VD (42.3%), FTD (40.2%), and AD (33.2%). Age and disease severity were associated with an increase of motor dysfunction (*p* < 0.001) during quarantine, whereas full physical autonomy before quarantine was a positive predictor against worsening in motor functions (OR 0.49 [95% CI 0.37–0.64], *p* < 0.001).

The increased prevalence of cognitive, behavioral and motor symptoms was not influenced either by regional distribution of different CDCD or by the regional prevalence of COVID-19 at the time of the study.

## Discussion

Our study shows that quarantine due to COVID-19 pandemic is associated with a dramatic increase in clinical symptoms of patients with dementia. According to family caregivers, social isolation and physical restraint caused a worsening in cognitive function, an aggravation of several behavioral symptoms, and a worsening in motor function. These effects were observed in all forms of dementia, but patients with DLB and AD showed the highest increase in cognitive and behavioral symptoms. In addition, the quarantine period was associated with an increase of caregiver’s burden, mainly characterized by anxiety.

To the best of our knowledge, this is the first nation-wide study that investigated the effects of quarantine in patients with dementia. Our data confirm the results of a previous, small study that investigated the effects of COVID-19 outbreak in patients with MCI and dementia (Canevelli et al., 2020). To evaluate factors associated with the quarantine-related clinical worsening, we performed extensive statistical analyses, and the most consistent relation was observed with the pre-pandemic physical status. Patients still able to walk unassisted or accompanied, after an acute interruption of physical activity, showed the greater worsening in clinical symptoms. Sex, age, duration of the disease, and disease severity had small and inconsistent effects. Several studies showed that physical activity has a positive impact on cognition of elderly adults (Fratiglioni et al., 2004; Bherer, 2015; Forbes et al., 2015; Young et al., 2015) and provided evidence that in patients with dementia there is an inverse relationship between physical activity, abnormal behavior, and cognitive decline (Nelson and Tabet, 2015; Karssemeijer et al., 2017). Patients physically more independent and, probably, with a more active life, enriched with a variety of social contacts and cognitive stimulation, are those most affected by the negative effects of confinement. Our study highlights the importance of maintaining physical activity in patients with dementia, and suggests the need for additional investigations to better understand this phenomenon.

Our study showed that 40% of the examined patients were totally aware of the COVID-19 pandemic and of social and physical restrictive measures planned to control virus transmission. Intriguingly, we found that patients with dementia still able to completely understand the need of quarantine, and therefore able to adopt adequate strategies to face the stress, had a significantly lower impairment in cognitive, behavioral and motor function. Contrariwise, less aware patients failed in creating new adaptive procedures to quarantine. Awareness of quarantine varied significantly among dementia subgroups and DLB patients showed the highest degree of partial and total degree of awareness. Taken together, the results of our study suggested that DLB patients are the most vulnerable to acute stress among the patients with dementia.

The neurobiological basis of the observed phenomenon has never been investigated. Examining subgroups of patients with dementia, we found an increase mainly of the core symptoms that characterize different types of dementing illness. This finding suggests that the observed phenomenon is not disease-specific but is more likely attributable to stress-induced changes. Studies in experimental animals showed that exposure to both acute and chronic stress evokes chemical changes in brain that impair the higher cognitive functions (Arnsten et al., 1999; Yamada et al., 2003). Uncontrollable, acute stress induces a diffuse increase in brain glucocorticoids and a specific catecholamine release in prefrontal cortex, impairing spatial memory tasks (Arnsten, 2015; Bahtiyar et al., 2020). Intriguingly, in animal models of acute physical and social restraints, an impairment of serotonin metabolism in the central nervous system was reported, with an alteration of immune response (Medina-Martel et al., 2013). In humans, stress drives several diseases, including cognitive disorders such as AD (Canet et al., 2019). Acute stress induces a neuroinflammatory response characterized by the release of several pro-inflammatory molecules and microglial activation that is a common feature of several neurodegenerative diseases (Sharma and Kanneganti, 2016). Finally, worsening of sleep, frequently reported in our study, is an additional explanation of our findings. Poor sleep is strongly associated with risk of multiple types of dementia and, via modulation of β-amyloid secretion, is directly involved in AD pathogenesis (Vanderheyden et al., 2018). Further studies are warranted to better elucidate the neurobiological mechanisms underlying the reported worsening of patients with dementia during quarantine and to provide therapeutic strategies.

Caregiving of dementia patients is associated with a higher prevalence of depressive and anxiety disorders, and impairments in physical health. Data from our study clearly showed that, as previously observed in the general population after SARS and Ebola outbreaks, quarantine is associated with increase in anxiety and depression. New health policies should be planned in the post-COVID-19 era to help family caregivers in delivering effective care. Finally, dementia Centers need to implement new strategies, as telemedicine (Goodman-Casanova et al., 2020; Hollander and Carr, 2020), in order to assist frailty people and support family members.

Some limitations of our study should be acknowledged. We examined dementia patients living at home and our data cannot be generalized to institutionalized patients with dementia. Then, we performed a cross-sectional study and, at present, it is unclear whether the observed clinical worsening is a transient or long-lasting phenomenon. Finally, all the clinical data regarding patient’s symptoms were collected from family caregivers as, due to quarantine rules, it was not possible to administer face-to-face standardized neuropsychological tests. However, previous studies showed that BPSD can be adequately evaluated by family caregivers and demonstrated that symptoms reported can be used in the clinical staging of dementia (Kwok et al., 2011; Yuan et al., 2020). Therefore, in order to evaluate if there is a time-effect, we have planned a new follow-up interview to the same caregivers. Finally, the major strength of our study is the high number of caregivers that have been interviewed in a relatively small amount of time and their reliability, having this role from the onset of dementia.

## Conclusion

In conclusion, interviewing family caregivers, we investigated for the first time the acute effects of quarantine in a large Italian population of patients with dementia, and we found that restrictive public health measures, are associated with an increase of dementia symptoms. These findings are important from a health policy perspective and suggest the need of new health care strategies to target patients with dementia during pandemic.

## Data Availability Statement

The raw data supporting the conclusions of this article will be made available by the authors, without undue reservation.

## Ethics Statement

The studies involving human participants were reviewed and approved by Comitato Etico Interaziendale di Torino. The patients/participants provided their written informed consent to participate in this study.

## Author Contributions

IR, AB, CM, AC, LB, CC, and VL designed the study and planned center recruitment. AC, CM, ER, and AB wrote the report. RDL and PP did the statistical analyses. ER, AV, VI, NV, FA, IA, PC, CB, RS, DQ, VG, GL, MF, GT, and CF contributed to the interpretation and discussion of results and reviewed the manuscript. The collaborating authors contributed to the collection of clinical data. All the authors and the collaborating authors contributed to the article and approved the submitted version.

## Conflict of Interest

The authors declare that the research was conducted in the absence of any commercial or financial relationships that could be construed as a potential conflict of interest.

## Appendix

### List of Collaborating Authors in the SINdem COVID-19 Study Group

**Aging Brain and Memory Clinic, Department of Neuroscience, University of Torino, Turin, Italy** (Erica Gallo, MD, erica.gallo@unito.it; Alberto Grassini, MD, alberto.grassini@unito.it; Andrea Marcinnò, MD, andrea.marcinno@unito.it; Fausto Roveta, MD, fausto.roveta@unito.it; Paola De Martino, MD, paolademartino@unito.it)
**Regional Neurogenetic Centre ASP-CZ Catanzaro, Catanzaro, Italy** (Francesca Frangipane, MD, francesca.frangipane@libero.it; Gianfranco Puccio, MD, puccio@arn.it; Rosanna Colao, MD, ros.colao@gmail.com; Maria Mirabelli, PsyD, mariamirabelli@libero.it)
**Memory Clinic, Fondazione Policlinico Agostino Gemelli, IRCCS Universitá Cattolica del Sacro Cuore, Rome, Italy** (Chiara Terracciano, MD, chiara.terracciano@uniroma2.it; Federica Lino, PsyD, federica.lino.psy@gmail.com)
**Department of Neuroscience, University of Padua, Padua, Italy** (Stefano Mozzetta, MD, st.mozzetta@gmail.com; Gianmarco Gazzola, MD, gianmarco.gazzola@gmail.com)
**CDCD Aulss6 Alta Padovana, Padua, Italy** (Giulia Camporese, MD, giulia.camporese@aulss6.veneto.it)
**Department of Biotechnological and Applied Clinical Sciences, Neurological Institute, University of L’Aquila, L’Aquila, Italy** (Simona Sacco, MD, simona.sacco@univaq.it)
**Avezzano Hospital, Avezzano, Italy** (Maria Carmela Lechiara, MD, mlechiara@asl1abruzzo.it)
**Department of Neuroscience, Imaging and Clinical Sciences, University G. d’Annunzio, Chieti, Italy** (Claudia Carrarini, MD, claudia.carrarini@live.it; Mirella Russo, MD, mirella.russo92@gmail.com)
**UOC Neurology, G. Mazzini Hospital, Teramo, Italy** (Alfonsina Casa lena, MD, alfonsinacasalena@yahoo.it)
**Clinica Neurologica San Salvatore Hospital, L’Aquila, Italy** (Patrizia Sucapane, MD, p_sucapane@yahoo.com)
**Division of Neurology, Scientific Institute for Research, Hospitalization, and Care (IRCCS), Foundation “Carlo Besta” Neurological Institute, Milan, Italy** (Pietro Tiraboschi, MD, Pietro.Tiraboschi@istituto-besta.it; Paola Caroppo, MD, paola.caroppo@istituto-besta.it; Veronica Redaelli, MD, veronica.redaelli@istituto-besta.it; Giuseppe Di Fede, MD, giuseppe.difede@istituto-besta.it)
**CDCD Serra Spiga ASP Cosenza, Cosenza, Italy** (Daniela Coppa, MD, dncoppa@gmail.com; Lenino Peluso, MD, dott.pelusolenino@gmail.com)
**CDCD Polistena Laureana ASP Reggio Calabria, Cinquefrondi, Italy** (Pasqualina Insarda, MD, linainsarda@tiscali.it)
**CDCD Jonio Sud District ASP Cosenza, Corigliano-Rossano, Italy** (Matteo De Bartolo, MD, debartolo.matteo@libero.it)
**First Division of Neurology, University of Campania “Luigi Vanvitelli”, Napoli, Napoli, Italy** (Sabrina Esposito, MD, sabrina.esposito1@unicampania.it)
**CDCD AORN “Ospedale dei Colli” – CTO, Napoli, Napoli, Italy** (Alessandro Iavarone, MD, alessandro.iavarone@ospedalideicolli.it)
**ASL Napoli 3 Sud, Napoli, Italy** (Carmine Fuschillo, MD, carmine.fuschillo@gmail.com)
**CDCD Neurologia, University of Campania “Federico II”, Napoli, Italy** (Elena Salvatore, MD, e.salvatore@unina.it; Chiara Criscuolo, MD, sky569@hotmail.com)
**IRCCS Istituto delle Scienze Neurologiche di Bologna, UOC Clinica Neurologica Rete Neurologica Metropolitana (NEUROMET), Italy** (Luisa Sambati, MD, luisasambati@gmail.com; Rossella Santoro, PhD, rossella.santoro@aosp.bo.it)
**Department of Neuroscience, Neurology Unit, AOU Sant’Anna di Icona – Ferrara, Ferrara, Italy** (Daniela Gragnaniello, MD, d.gragnaniello@ospfe.it)
**CDCD Ospedale del Delta, AUSL Ferrara, Ferrara, Italy** (Ilaria Pedriali, MD, ilaria.pedriali@ospfe.it)
**CDCD, AUSL of Parma, Parma, Italy** (Livia Ludovico, MD, lludovico@ausl.pr.it)
**AUO Policlinico Modena, Modena, Italy** (Annalisa Chiari, MD, chiari.annalisa@aou.mo.it)
**UOC Cognitive Disorders and Dementia, Department of Primary Care, AUSL Modena, Italy** (Andrea Fabbo, MD, a.fabbo@ausl.mo.it; Petra Bevilacqua, PsyD, p.bevilacqua@ausl.mo.it; Chiara Galli, PsyD, ch.galli@ausl.mo.it; Silvia Magarelli, PsyD, s.magarelli@ausl.mo.it)
**Fondazione Santa Lucia IRCCS, Roma, Italy and Menninger Department of Psychiatry and Behavioural Sciences, Baylor College of Medicine, Houston, TX, United States** (Gianfranco Spalletta, MD, g.spalletta@hsantalucia.it)
**Fondazione Santa Lucia IRCCS, Roma, Italy** (Nerisa Banaj, MD, n.banaj@hsantalucia.it; Giulia Caruso, PsyD, g.caruso@hsantalucia.it)
**Fondazione Santa Lucia IRCCS, Roma, and Dipartimento di Neuroscienze, Università di Roma “Tor Vergata”, Roma, Italy** (Desirée Estela Porcari, PsyD, de.porcari@hsantalucia.it)
**AOU Sant’Andrea, Roma, Italy** (Franco Giubilei, MD, franco.giubilei@uniroma1.it)
**AO San Giovanni Addolorata, Roma, Italy** (Anna Rosa Casini, MD, arosa.casini@virgilio.it)
**Campus Biomedico, University of Roma, Roma, Italy** (Francesca Ursini, MD, f.ursini@unicampus.it)
**Department of Neuroscience, University of Roma “La Sapienza”, Roma, Italy** (Giuseppe Bruno, MD, giuseppe.bruno@uniroma1.it)
**Department of Geriatrics, Fondazione Poliambulanza di Brescia, Italy** (Stefano Boffelli, MD, stefano.boffelli@poliambulanza.it)
**Luigi Sacco Hospital, University of Milano, Milano, Italy** (Michela Brambilla, PsyD, michela.brambilla@libero.it)
**Unit of Neurology, IRCCS San Raffaele Scientific Institute and Vita-Salute San Raffaele University, Milan, Italy** (Giuseppe Magnani, MD, magnani.giuseppe@hsr.it; Francesca Caso, MD, caso.francesca@hsr.it; Edoardo G. Spinelli, MD, spinelli.edoardogioele@hsr.it)
**Unit of Behavioral Neurology IRCCS Mondino Foundation, and Department of Brain and Behavioral Sciences, University of Pavia, Italy** (Elena Sinforiani, MD, elena.sinforiani@mondino.it; Alfredo Costa, MD, alfredo.costa@mondino.it)
**Department of Experimental and Clinical Medicine, Polytechnic University of Marche, Ancona, Italy** (Simona Luzzi, MD, s.luzzi@staff.univpm.it)
**CDCD Mazzoni Hospital, Ascoli Piceno, Italy** (Gabriella Cacchiò, MD, cacchiogabriella@tiscali.it)
**A.I.M.A. –sez Parma** (Marta Perini, PsyD, perini_marta@libero.it)
**CDCD Area Vasta 4, Fermo, Italy** (Rossano Angeloni, MD, rossano.angeloni@sanita.marche.it)
**Geriatric Operative Unit, IRCCS-INRCA, Fermo, Italy** (Cinzia Giuli, PsyD, c.giuli@inrca.it)
**CDCD Area Vasta 3, Macerata, Italy** (Katia Fabi, MD, katia.fabi@libero.it)
**Azienda Ospedaliera Marche Nord, Pesaro, Italy** (Marco Guidi, MD, marcoguidi55@gmail.com)
**CDCD San Benedetto del Tronto, Italy** (Cristina Paci, MD, cpaci@libero.it)
**CDCD IRCSS Neuromed di Pozzilli, Isernia, Italy** (Annaelisa Castellano, MD, annaelisacastellano@yahoo.it)
**Department of Neuroscience, Neurology Division, OORR Foggia, Italy** (Elena Carapelle, MD, elecarpi@hotmail.it)
**Neurodegenerative Centre, University of Bari “Aldo Moro”, Bari, Italy** (Rossella Petrucci, RN, rpetrucci78@gmail.com; Miriam Accogli, ScD, miriam.accogli@gmail.com)
**CDCD DSS of Campi Salentina, Lecce, Italy** (Giovanna Nicoletta Trevisi, MD, trevisigiovanna@libero.it)
**CDCD DSS of Lecce, Lecce, Italy** (Serena Renna, MD, tosen@tin.it)
**CDCD DSS of Maglie, Maglie, Italy** (Antonella Vasquez Giuliano, MD, antonellavasquezgiuliano@gmail.com)
**Department of Neurology, University of Milano – Bicocca, Milano, Italy** (Fulvio Da Re, MD, darefulvio@gmail.com)
**CDCD PO Santissima Trinità, ASSL Cagliari, Cagliari, Italy** (Antonio Milia, MD, antoniomilia55@gmail.com; Giuseppina Pilia, MD, giusi.pilia77@gmail.com; Maria Giuseppina Mascia, MD, mgmascia@gmail.com)
**CDCD Area Vasta 1, Cagliari, Italy** (Valeria Putzu, MD, putzu.valeria@tiscali.it)
**Section of Neurology, Department of Biomedicine, Neuroscience and Advanced Diagnostics, University of Palermo, Palermo, Italy** (Tommaso Piccoli, MD, tommaso.piccoli@gmail.com; Luca Cuffaro, MD, cuffaro.luca@gmail.com; Roberto Monastero, MD, roberto.monastero@gmail.com)
**Neurology and Neurophysiopathology Unit, AOUP “Paolo Giaccone”, Palermo, Italy** (Antonella Battaglia, PsyD, antobatt1994@yahoo.it; Valeria Blandino, PsyD, valeriabl@libero.it; Federica Lupo, PsyD, federicalupo1@gmail.com)
**UO Neurodegenerative Disorders, ASP 2, Caltanisetta, Italy** (Eduardo Cumbo, MD, eduardo.cumbo@tiscali.it)
**Department “G.F. Ingrassia”, University of Catania, Catania, Italy** (Antonina Luca, MD, antolucaster@gmail.com)
**AO Cannizzaro, Catania, Italy** (Giuseppe Caravaglios, MD, giuseppe.caravaglios@gmail.com)
**Psychogeriatric Unit, ASP Messina, Messina, Italy** (Annalisa Vezzosi, MD, psicogeriatria@asp.messina.it)
**Neurology I, Department of Neuroscience, Psychology, Drug Research and Child Health, AOU Careggi, Firenze, Italy** (Valentina Bessi, MD, valentina.bessi@unifi.it)
**CDCD, Neurology I, AOU University of Pisa, Pisa, Italy** (Gloria Tognoni, MD, gloria.tognoni@med.unipi.it)
**Geriatric Unit, Department of Clinical and Experimental Medicine, University of Pisa, Pisa, Italy** (Valeria Calsolaro, MD, valina82@gmail.com)
**AOU Careggi and University of Florence** (Giulia Lucarelli, MD, giulialucarelli31@gmail.com)
**CDCD Territoriale, USL Umbria 1, Perugia, Italy** (Serena Amici, MD, serena.amici@uslumbria1.it; Alberto Trequattrini, MD, alberto.trequattrini@uslumbria1.it; Salvatore Pezzuto, MD, salvatore.pezzuto@uslumbria1.it)
**Department of Medicine, University of Perugia, Perugia, Italy** (Patrizia Mecocci, MD, patrizia.mecocci@unipg.it; Giulia Caironi, MD, giulia.caironi@hotmail.it)
**CDCD AUSSL 7 Pedemontana, Bassano del Grappa, Italy** (Barbara Boselli, MD, CDC@aulss7.veneto.it)
**CDCD Geriatria, Dolo, Venezia, Italy** (Marino Formilan, MD, uva.geriatriadolo@aulss3.veneto.it)
**CDCD Geriatric Unit, University of Padua, Padua, Italy** (Alessandra Coin, MD, alessandra.coin@unipd.it)
**CDCD AULSS 9 Scaligera, Verona, Italy** (Laura De Togni, MD, laura.detogni@aulss9.veneto.it; Francesca Sala, MD, francesca.sala@aulss9.veneto.it; Giulia Sandri, MD, neuropsicologia.villafranca@aulss9.veneto.it)
**CDCD AULSS 2 Marca Trevigiana, Treviso, Italy** (Maurizio Gallucci, MD, maurizio.gallucci@aulss2.veneto.it; Anna Paola Mazzarolo, PhD, annapaola.mazzarolo@aulss2.veneto.it; Cristina Bergamelli, PhD, cristina.bergamelli@aulss2.veneto.it)
**ASST Grande Ospedale Metropolitano, Niguarda, Milano, Italy** (Serena Passoni, MD, serena.passonio@spedaleniguarda.it)
