# Supplementary material for: The Impact of COVID-19 Quarantine on Patients With Dementia and Family Caregivers: A Nation-Wide Survey
Source: Front Aging Neurosci. 2021 Jan 18;12:625781. doi: 10.3389/fnagi.2020.625781 (PMC7849158; doi:10.3389/fnagi.2020.625781)
Supplement: Supplementary file 1 [file Table_1.DOCX]

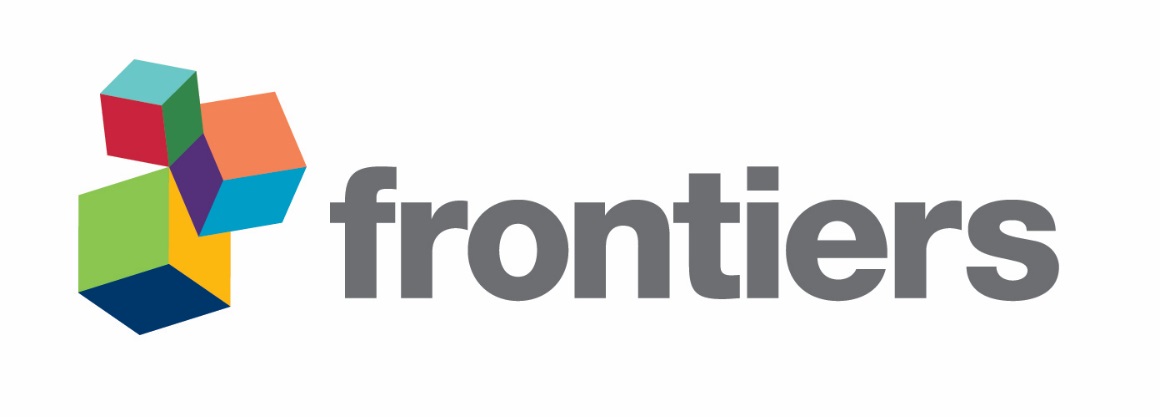


Supplementary Material

# Supplementary Data

**1. Clinical criteria for the diagnosis of different subtypes of dementia pag. 2**

**2. COVIDem and dementia – Survey Form A pag. 5**

**3. COVIDem and dementia – Survey Form B pag. 11**

**The impact of COVID-19 quarantine on patients with dementia and family caregivers: a nation-wide survey**

Innocenzo Rainero, Amalia C. Bruni, Camillo Marra, Annachiara Cagnin, Laura Bonanni, Chiara Cupidi, Valentina Laganà, Elisa Rubino, Alessandro Vacca, Raffaele Di Lorenzo, Paolo Provero, Valeria Isella, Nicola Vanacore, Federica Agosta, Ildebrando Appollonio, Paolo Caffarra, Cinzia Bussè, Renato Sambati, Davide Quaranta, Valeria Guglielmi, Giancarlo Logroscino, Massimo Filippi, Gioacchino Tedeschi, Carlo Ferrarese, and the SINdem COVID-19 Study Group

**METHODS**

**Centers and participants enrollment**

*Diagnostic criteria*

1. Alzheimer’s disease: National Institute on Aging-Alzheimer's Association Workgroups on Diagnostic Guidelines for Alzheimer's Disease.^1^
2. Dementia with Lewy Bodies: Fourth Consensus Report of the DLB Consortium.^2^
3. Frontotemporal dementias: Behavioral Variant Frontotemporal dementia – Frontotemporal Dementia Consensus (FTDC) criteria,^3^ Semantic Variant Primary Progressive Aphasia and Nonfluent/agrammatic Primary Progressive Aphasia – Gorno-Tempini et al. criteria.^4^
4. Vascular dementia: Diagnostic Criteria for Vascular Cognitive Disorders: A VASCOG Statement.^5^

**References**

1. McKhann GM, Knopman DS, Chertkow H, Hyman BT, Jack CR Jr, Kawas CH, Klunk WE, Koroshetz WJ, Manly JJ, Mayeux R, Mohs RC, Morris JC, Rossor MN, Scheltens P, Carrillo MC, Thies B, Weintraub S, Phelps CH.McKhann GM, et al. [The diagnosis of dementia due to Alzheimer's disease: recommendations from the National Institute on Aging-Alzheimer's Association workgroups on diagnostic guidelines for Alzheimer's disease.](https://pubmed.ncbi.nlm.nih.gov/21514250/?from_term=Alzheiemr+disease+NIA+criteria&from_exact_term=alzheimer+disease+nia+criteria&from_pos=1) *Alzheimers Dement* 2011;7:263-269.
2. McKeith IG, Boeve BF, Dickson DW, Halliday G, Taylor JP, Weintraub D, Aarsland D, Galvin J, Attems J, Ballard CG, Bayston A, Beach TG, Blanc F, Bohnen N, Bonanni L, Bras J, Brundin P, Burn D, Chen-Plotkin A, Duda JE, El-Agnaf O, Feldman H, Ferman TJ, Ffytche D, Fujishiro H, Galasko D, Goldman JG, Gomperts SN, Graff-Radford NR, Honig LS, Iranzo A, Kantarci K, Kaufer D, Kukull W, Lee VMY, Leverenz JB, Lewis S, Lippa C, Lunde A, Masellis M, Masliah E, McLean P, Mollenhauer B, Montine TJ, Moreno E, Mori E, Murray M, O'Brien JT, Orimo S, Postuma RB, Ramaswamy S, Ross OA, Salmon DP, Singleton A, Taylor A, Thomas A, Tiraboschi P, Toledo JB, Trojanowski JQ, Tsuang D, Walker Z, Yamada M, Kosaka K.McKeith IG, et al. Diagnosis and management of dementia with Lewy bodies: Fourth consensus report of the DLB Consortium. *Neurology* 2017;89:88-100.
3. Rascovsky K, Hodges JR, Knopman D, Mendez MF, Kramer JH, Neuhaus J, van Swieten JC, Seelaar H, Dopper EG, Onyike CU, Hillis AE, Josephs KA, Boeve BF, Kertesz A, Seeley WW, Rankin KP, Johnson JK, Gorno-Tempini ML, Rosen H, Prioleau-Latham CE, Lee A, Kipps CM, Lillo P, Piguet O, Rohrer JD, Rossor MN, Warren JD, Fox NC, Galasko D, Salmon DP, Black SE, Mesulam M, Weintraub S, Dickerson BC, Diehl-Schmid J, Pasquier F, Deramecourt V, Lebert F, Pijnenburg Y, Chow TW, Manes F, Grafman J, Cappa SF, Freedman M, Grossman M, Miller BL. Rascovsky K, et al. Sensitivity of revised diagnostic criteria for the behavioural variant of frontotemporal dementia. *Brain* 2011;134:2456-2477.
4. Gorno-Tempini ML, Hillis AE, Weintraub S, Kertesz A, Mendez M, Cappa SF, Grossman M. Classification of primary progressive aphasia and its variants. *Neurology* 2011;76:1006–1014.
5. Sachdev P, Kalaria R, O'Brien J, Skoog I, Alladi S, Black SE, Blacker D, Blazer DG, Chen C, Chui H, Ganguli M, Jellinger K, Jeste DV, Pasquier F, Paulsen J, Prins N, Rockwood K, Roman G, Scheltens P; Internationlal Society for Vascular Behavioral and Cognitive Disorders, et al. Diagnostic criteria for vascular cognitive disorders: a VASCOG statement. *Alzheimer Dis Assoc Disord* 2014;28(3):206-218.

.

2. COVID-19 and dementia – Form A

Form A

**A.1 Caregiver**

Sex: Male ___ Female ___

Age ________ Education (years)________

Relative of the person with dementia (type):____________________________________________

- Do you live with the person suffering from dementia?

YES_____

NO_____

- If so, were you already living together before quarantine?

YES_____

NO_____

- If not, is the patient institutionalized?

YES_____

NO_____

Have you the possibility to see him regularly

YES____

NO____

- Are there other people in the house?

YES_____

NO_____

- Are you working during quarentine?

YES_____

NO_____

- Has your life changed with quarentine?

NO

YES, I have less time to devote to myself.

YES, I have relationship problems with other family members.

7) Has the relationship with your relative changed?

NO

YES, increase of conflicts

YES, improvement of the relationship

8) Are you concerned about possible consequences of COVID 19 on the patient's health?

YES_____

NO_____

9) Do you think that specific guidance on the prevention of COVID 19 infection in patients with dementia may be useful?

YES_____

NO_____

10) In general, are you experiencing problems in care/therapeutic continuity?

YES_____

NO_____

11) Have you been in the situation to search first aid services (out-of-hours service doctor/ emergency medical care) for yourself or the patient due to the lack of assistance on the territory by the medical practitioner (family doctor) or specialist consultant?

YES_____

NO_____

12) Would you be in favour of the use of tools such as telephones and computers, and use of remote psychological support for outpatient visits in suspended services?

YES_____

NO_____

13) Did you receive any help or support during the COVID emergency?

NO

Yes, by the family

Yes, from the neighbours/friends

Yes, from the Municipality

Yes, from volunteering (associations, parishes, red cross, others)

14) Did you feel isolated or abandoned during the COVID emergency?

YES_____

NO_____

15 Did you feel overwhelmed or helpless?

YES_____

NO_____

16 Did you present

anguish Yes ___No___

depression Yes ___No___

anxiety Yes ___No___

irritability Yes ___No___

**A.2 Patient**

Age__________________

Sex: Male ___ Female ___

Type of dementia: ____________________________________________________________

Year of dementia onset____________

Quarantine start date: ___ / ___ / ___

1. Before quarantine the patient was used to get out of the house

Yes, independently ___

Yes, accompanied, ___

No ___

2) What is the stage of the disease?

Mild

Moderate

Severe

Bedridden

3) Have you noticed patient’s behavioural changes since the beginning of the quarantine?

YES_____

NO_____

If Yes, what are the behavioral symptoms that have increased in intensity but were already present before quarantine?

o Apathy (reduction of initiative)

o Anxiety

o Depression

o Sleep disorders

o Delusions (i.e. the patient has thoughts that someone wants to hurt him, steal from him, kill him, etc.).

o Hallucinations (the patient sees or hears things or people that are not there)

o Irritability

o Aggressiveness

o Wandering (the patient walks around aimlessly and irrepressibly)

o Agitation

o Change in appetite

4) Did you notice any new behavioral symptoms, which were not present before the quarantine period?

o There were no new symptoms detected

o Apathy (reduction of initiative)

o Anxiety

o Depression

o Sleep disorders

o Delusions (the patient has thoughts that someone wants to hurt him, wants to rob him, kill him etc.)

o Hallucinations (seeing and hearing things or people who are not there)

o Irritability

o Aggressiveness

o Wandering (walking around aimlessly and irrepressibly)

o Agitation

o Change in appetite

5) Because of these disorders, was it necessary to change the medication therapy?

YES_____

NO_____

6) Have you noticed any changes in the patient’s motor performances in the last month?

o I think he/she walks better than before

o No

o He/she walks slower

o He/she can't stand up independently from the chair anymore.

o He/she doesn't get up from bed anymore

7) Have you noticed any changes in the patient’s cognitive symptoms that were present before quarantine?

NO_______

YES______

o Increased confusion

o Greater forgetfulness

o More difficulty in finding the correct words

o In-door space disorientation and difficulties of house recognition

o Disorientation in time

o Loss of recognition of family members

o Troubles of self-recognition in the mirror

8) Do you think the disease has progressed faster during the quarantine period?

YES_____

NO, I think it's taking its natural course______

9) Is the patient aware of the COVID emergency?

YES_____

PARTIALLY ____

NO_____

Date .......................................................................................

3. COVID-19 and dementia – Form B

**FORM B**

A1. DESCRIPTION of CDCD (centres for cognitive decline and dementia)

1. Region of the CDCD _______________________________________________________

2. The centre is: university clinic ___ hospital clinic ___ local service___

3. On average, how many outpatient visits of patients with dementia per month are made (refer to the period before COVID pandemic)? ________

4. Is psychological counselling available for caregivers?

YES_____

NO_____

5. Is a social worker available?

YES_____

NO_____

A.2 CHANGE IN SERVICES PROVIDED BY THE CDCD DUE TO QUARANTINE

1. Has normal outpatient activity been suspended?

YES, since__________________________

NO, the activity has remained unchanged

NO, but the activity has been greatly reduced

2. Has hospitalisation been suspended?

YES, since__________________________

NO_____

3. Has teleconsultation visit been activated? Yes ___ No ___

YES_____

NO_____

4. the deadline of therapeutic schedules have been automatically extended beyond the regulatory rules of the Region?

YES_____

NO_____

5. Has the caregiver support service been activated?

YES_____

NO_____

6. Is there a way to renew the therapeutic schedules through e-mail after telephone consultation with caregivers?

YES_____

NO_____

7. Is there the possibility of urgent visits for cognitive deficit or behavioural disorders?

YES_____

NO_____

8. Have drug trials (profit and non-profit) been suspended?

YES_____

NO_____

9. Have the semi-residential and residential services you manage (Day Centre, Alzheimer's Café) been suspended?

YES_____

NO_____

Date ________________________________________________.
